# The Dopamine Receptor D4 Gene (*DRD4*) and Financial Risk-Taking: Stimulating and Instrumental Risk-Taking Propensity and Motivation to Engage in Investment Activity

**DOI:** 10.3389/fnbeh.2018.00034

**Published:** 2018-03-02

**Authors:** Rafał Muda, Mariusz Kicia, Małgorzata Michalak-Wojnowska, Michał Ginszt, Agata Filip, Piotr Gawda, Piotr Majcher

**Affiliations:** ^1^Faculty of Economics, Maria Curie-Sklodowska University, Lublin, Poland; ^2^Department of Cancer Genetics with Cytogenetics Laboratory, Medical University of Lublin, Lublin, Poland; ^3^Department of Rehabilitation and Physiotherapy, Medical University of Lublin, Lublin, Poland

**Keywords:** *DRD4* gene, financial risk-taking, investors, dopamine, genetic determinants, risk preferences

## Abstract

The Dopamine receptor D4 gene (DRD4) has been previously linked to financial risk-taking propensity. Past works demonstrated that individuals with a specific variant of the DRD4 gene (7R+) are more risk-seeking than people without it (7R−). The most prominent explanation for this effect is the fact that 7R+ individuals are less sensitive to dopamine and thus seek more stimulation to generate “normal” dopaminergic activity and feel pleasure. However, results about this relationship have not been conclusive, and some revealed a lack of the relationship. In the current work, we tested if those unclear results might be explained by the motivation that underlies the risk-taking activity; i.e., if people take risks to feel excitement or if they take risk to obtain a specific goal. In our study we tested the differences in risk-taking between 7R+ and 7R− among people who are experienced in financial risk-taking (113 investors) and non-experienced financial decision makers (104 non-investors). We measured risk-taking propensity with the Holt-Laury test and the Stimulating-Instrumental Risk Inventory. Moreover, we asked investors about their motivations for engaging in investment activity. Our study is the next one to report a lack of differences in risk-taking between 7R+ and 7R− individuals. As well, our results did not indicate any differences between the 7R+ and 7R− investors in motivation to engage in investment activity. We only observed that risk-taking propensity was higher among investors than non-investors and this was noticed for all measures. More research is needed to better understand the genetic foundations of risk-taking, which could answer the question about the substantial variation in the domain of risky financial decisions.

## Introduction

As previous studies have indicated, the dopamine gene *Dopamine Receptor D4 Gene (DRD4)* is one of the most promising candidates that can be associated with risk-taking propensity (Carpenter et al., 2011; Dreber et al., 2011). The *DRD4*, a dopamine D4 receptor gene, is located near the telomere of chromosome 11p and contains a 48-bp Variable Number Tandem Repeat (VNTR) polymorphism in the third exon, repeated between 2 and 11 times (Grady et al., 2003). Moreover, the 48-bp repeat is thought to reside in the third cytoplasmic loop of the receptor protein and seems to affect the function of the D4 receptor (Ptácek et al., 2011). It was discovered that a variant with 7 or more VNTR repeats (7R+) is connected with the decreased binding of dopamine (Asghari et al., 1995). 7R+ individuals are less sensitive to dopamine and thus require a higher level of stimuli to produce a similar response as compared with people with the 7R− variant (with less than 7 VNTR repeats; Schoots and Van Tol, 2003). The site of dopamine’s release seems to determine the role that it plays. Four major dopamine-rich pathways have been identified within the brain (mesolimbic, mesocortical, nigrostriatal, and tuberoinfundibular pathways). These pathways arise from two regions of the midbrain: the ventral tegmental area (VTA) and the substantia nigra, which primarily projects to the striatal complex—ventral striatum (VS) and dorsal striatum (Ernst and Luciana, 2015). Several studies have shown that dopaminergic projection from the VTA to the VS is particularly important in reward processing (McBride et al., 1999; Pierce and Kumaresan, 2006).

As a gene responsible for the regulation of the dopaminergic system and in turn reward processing (Wise, 2002), the *DRD4* gene may contribute to the behaviors connected with dopamine levels, e.g., risk-taking. The role of dopamine in reward processing and risk taking has been investigated in animal studies. For example, rats with an over-expressed dopamine transporter showed increased impulsivity for smaller and sooner rewards, and increased risk proneness (Adriani et al., 2009). Moreover, release of dopamine reinforces particular behaviors (especially those related to the expectation of reward), causes feelings of joy, and increases physiological arousal (Berridge and Robinson, 1998). As Schwarz (2012) noticed, bodily experiences like physiological arousal might inform us about physical states of the organism that, in turn, may be perceived as a source of information and influence decision-making. Moreover, through the activation of the nucleus accumbens, which is activated during the anticipation of monetary gains and positively correlates with a positive affect, dopamine is related to risk-taking behavior (Kuhnen and Knutson, 2005). Taking this into account, we should expect that the *DRD4* gene plays a moderating role in risk-taking propensity and 7R+ individuals should take more risks.

Indeed, previous studies about behavioral traits and the *DRD4* gene revealed that 7R+ individuals are prone to take more risks in specific situations that may cause positive stimulation, i.e., gambling or drinking alcohol. Researchers indicated that the presence of the 7R allele is connected to alcoholism (Laucht et al., 2007), impulsivity (Eisenberg et al., 2007), pathological gambling (Pérez de Castro et al., 1997), or novelty-seeking (Ebstein et al., 1996).

Also in the domain of financial risk-taking, so far, four studies have revealed that 7R+ individuals make more risky decisions than 7R− individuals (Dreber et al., 2009, 2011; Kuhnen and Chiao, 2009; Carpenter et al., 2011). More precisely, Dreber et al. (2009) showed that the 7R+ polymorphism is associated with higher financial risk-taking and explains roughly 20% of the variance in financial risk-taking. In their next article, Dreber et al. (2011) confirmed the previous result. However, they found that the 7R+ variant is related to higher risk-taking propensity only among men but not among women. Also, Kuhnen and Chiao (2009) noticed a significant relationship between 7R+ and risk taking—in their study, 7R+ individuals invested 25% more assets in risky options than 7R− individuals.

However, some findings revealed a lack of differences. Another four studies failed to find significant differences between 7R+ and 7R− individuals in the domain of financial risk-taking (Eisenegger et al., 2010; Frydman et al., 2011; Dreber et al., 2012; Anderson et al., 2015). For example, Frydman et al. (2011) asked subjects to make choices between 140 pairs of monetary gambles. In each pair, subjects decided if they preferred the certain non-negative option involving a payout of *x* with 100% chance or a risky option involving a gain $y and a loss $z with equal probability. The results revealed that 7R+ individuals chose risky options in 39% of cases, while 7R− chose risky options in 38% of cases. No differences were also shown between the group of 7R+ and 7R− individual investors in both financial risk-taking task (choices between a certain payoff ranging from $140 to $1000 and a 50:50 gamble between the gain of $1000 or nothing) and measures of equity holdings (based on national registry data on detailed asset holdings; Anderson et al., 2015). A lack of differences in risk-taking between 7R+ and 7R− was also observed in a group of owners, presidents and managers of large companies who performed the investment task. In this task participants started with $250 and decided how much money they allocated in a risky investment which gave a 50% chance to multiply the invested amount 2.5 times, and a 50% chance to lose the allocated amount (Dreber et al., 2012). Surprisingly, in two other studies that used the same investment task, differences in risk-taking between 7R+ and 7R− were observed (Dreber et al., 2009, 2011).

The aim of our study is to verify if the previous inconclusive results about the *DRD4* gene and financial risk-taking might be explained by different needs that motivate risk-taking behavior. In the financial domain, risky behaviors might depend on motives that stimulate risk-taking. We can distinguish two kinds of risk preference that could potentially moderate the association between *DRD4* gene and risk-taking: (1) stimulating; and (2) instrumental risk-taking (Zaleśkiewicz, 2001). The motivation behind stimulating risk is to take action due to need for excitement seeking and to provide positive emotional arousal. Such experiences motivate to seek stimuli that provide pleasant feelings, and thus one is more prone to engage in risky activities. On the other hand, instrumental risk-taking is driven by motives that are oriented on achieving a specific goal and analytic information processing instead of arousal seeking. For example, consider one who has $1000 and desperately needs an additional $1000 for medical treatment by the end of the day. After analyzing every possibility how to collect the money, one concludes that the only option is to play in the casino. Although, one engages in risky activity, this is due to a rational decision motivated by the need to achieve a particular economic goal (i.e., gain an additional $1000 for medical treatment), not due to the need for experiencing pleasant feelings connected with gambling (Zaleśkiewicz, 2001).

In our study, we want to test if the *DRD4* gene is connected with financial risk-taking propensity in general, or if it is associated only with a specific risk-taking propensity that is oriented toward the search for stimulation and arousal. Taking into account that: (1) 7R+ individuals are more prone to engage in risky behaviors that increase arousal (e.g., gambling or drinking alcohol), as well as; (2) they need more stimuli to overcome the blunted response to dopamine to function “normally”, we might expect that, in the financial domain, we will notice the differences between 7R+ and 7R− individuals in stimulating risk-taking propensity but not in instrumental risk-taking propensity.

Additionally, in our study, we wanted to test the differences in risk-taking between 7R+ and 7R− among people who are experienced in financial decision-making and risk-taking (i.e., stock market investors). So far, only three studies have focused on different groups than students (Dreber et al., 2011, 2012; Anderson et al., 2015), and testing such a group could give more reliable results than testing just undergraduate students. Moreover, as Dorn and Sengmueller (2009) revealed, investors who have a tendency to trade excessively (which implies higher costs and in turn increases the risk) report enjoying investing or gambling^1^. This result suggests that investors who enjoy investing are more prone to accept risk for other reasons than monetary incentives (e.g., looking for excitement). This seems to be in line with our hypothesis that people who seek stimulation (i.e., 7R+ individuals) might take more risks in the financial domain than others.

## Materials and Methods

### Participants

We conducted our study on two groups: (1) a group of private investors (*n* = 120, mean age = 33.63 [three subjects missing data for age], standard deviation [SD] = 9.85; we successfully genotyped 113 investors, mean age = 33.70 [one subject missing data for age], SD = 9.95, mean years of investing [missing data for four subjects] = 10.27, SD = 7.34, for 20 subjects investment activity was a main source of income, for 89 subjects it was additional income [missing data for four subjects]); and (2) a group of non-investors (*n* = 112, mean age = 32.46 SD = 10.14; we successfully genotyped 104 non-investors, mean age = 32.34 [missing data for age for one subject], SD = 10.00). We defined an investor/non-investor as a person who invests/has never invested assets in the stock market or allocates/has never allocated money in an investment found. Moreover, we controlled for academic major (financial/economics vs. others) and found no differences between group of investors and non-investors (*χ*^2^[1, *n* = 224] = 1.03, *p* = 0.348, *φ* = 0.068).

### Data Collection

The study was conducted during the Wall Street Conference—the biggest conference in Poland about the practice of investment, organized by the Society of Individual Investors. Before the event, all conference participants were informed about the study and invited to participate via email. Subjects were also recruited by flyers distributed at the conference place. For data collection, we invited subjects to a dedicated location in the conference place. The experiment was done with paper and pencil and tasks referred to non-incentivized decisions. At the beginning, we informed participants about the study protocol and collected their written consent to take part in the experiment. Next, we asked participants to provide two salivary samples. Cotton swab–derived buccal cells were scraped from the inner side of the cheeks. Prior to the sample collection, each of participants vigorously rinsed their mouth with water for about 30 s to remove food particles. They were given two cotton swabs and two test tubes labeled with a participant number. Then each of the participants was asked to give a buccal swab from each side of the cheek by scraping the inside of their cheek with the swab firmly for 30 s. Donors were reminded to turn the swabs to utilize both sides of the swab. In order to maximize the buccal cell yield, the samples were brought back to the laboratory in an ice-filled cooler. Afterward, subjects completed a sociodemographic survey and two risk-taking tasks.

### Risk-Taking Tasks

We measured the risk-taking propensity in three ways. The first one was the Holt-Laury test (Holt and Laury, 2002), which is one of the most widely used tests to measure risk-taking propensity in experimental economics. The Holt-Laury test is a measure based on choices between paired lotteries that involve only gains (see Table 1). In each pair (all pairs are presented in advance), the participant makes a decision between Lottery A and Lottery B. For each decision, lotteries give the possibility to win a fixed amount: Lottery A: 100 PLN or 80 PLN (which is about 25 USD and 20 USD), Lottery B: 185 PLN and 5 PLN. The subsequent lottery pairs differ on the probability of obtaining particular amount. In the first pair, the probability of winning the larger payoff (100 PLN and 185 PLN, respectively) is relatively low (i.e., a 10% chance), whereas the probability of winning a smaller payoff (i.e., 80 PLN and 5 PLN) is relatively high (i.e., a 90% chance). With each new pair, the probability of getting the higher reward increases by 10 percentage points, and in the last decision the chance for a higher gain is 100%.

**Table 1:** Choices between paired lotteries in the Holt-Laury test (Holt and Laury, 2002; polish version Tyszka, 2010).

Lottery A	Lottery B	Expected value of Lottery A	Expected value of Lottery B
10% 100 PLN; 90% 80 PLN	10% 185 PLN; 90% 5 PLN	82	23
20% 100 PLN; 80% 80 PLN	20% 185 PLN; 80% 5 PLN	84	41
30% 100 PLN; 70% 80 PLN	30% 185 PLN; 70% 5 PLN	86	59
40% 100 PLN; 60% 80 PLN	40% 185 PLN; 60% 5 PLN	88	77
**50% 100 PLN; 50% 80 PLN**	**50% 185 PLN; 50% 5 PLN**	**90**	**95**
60% 100 PLN; 40% 80 PLN	60% 185 PLN; 40% 5 PLN	92	113
70% 100 PLN; 30% 80 PLN	70% 185 PLN; 30% 5 PLN	94	131
80% 100 PLN; 20% 80 PLN	80% 185 PLN; 20% 5 PLN	96	149
90% 100 PLN; 10% 80 PLN	90% 185 PLN; 10% 5 PLN	98	167
100% 100 PLN; 0% 80 PLN	100% 185 PLN; 0% 5 PLN	100	185

*Bold text indicates the first lottery pair where expected value of Lottery B is higher than Lottery A. During the experiment, the text was not bolded and participants were presented only with first two columns and the place to indicate the response (columns with expected values were not presented)*.

Notice that the larger gain in Lottery B (i.e., 185 PLN) is higher than the larger gain in Lottery A (i.e., 100 PLN), whereas a smaller gain in Lottery A (i.e., 80 PLN) is larger than a smaller gain in Lottery B (i.e., 5 PLN). Thus, depending on the participant’s risk-taking propensity, the switch from Lottery A to Lottery B will occur at different points. Someone who is an extreme risk-seeker might decide to take a chance to win the highest payoff and choose Lottery B in the first step, whereas one who is extremely risk averse and does not want to risk “losing” a moderate payoff might choose Lottery A until the last step.

The next two risk-taking measures were stimulating and instrumental risk-taking. Both were from the Stimulating-Instrumental Risk Inventory (Zaleśkiewicz, 2001). The Stimulating-Instrumental Risk Inventory is a questionnaire composed of 17 questions: 10 questions measure stimulating risk-taking (e.g., I often take risk just for fun; Gambling seems something very exciting to me), and seven questions instrumental risk-taking (e.g., At work I would prefer a position with a high salary which could be lost easily to a stable position but with a lower salary). In the Stimulating-Instrumental Risk Inventory each statement is scored on a five-point scale with end-points described as 1—*does not describe me at all*; to 5—*describes me very well*.

Moreover, we asked private investors about their motivations for engaging in investment activity. Asset allocation in the stock market is a risky activity itself. Thus, by asking investors what motives underlay their decision to start investing, we wanted to test on the basis of real-life behavior the assumption that 7R+ individuals take more risk because of their need for stimuli. After the study, three independent judges evaluated the answers and grouped them into two categories: (1) the instrumental motivation category in which judges included all motives focused on achieving a specific goal, e.g., multiplying capital, saving for retirement; and (2) stimulating motivation category in which judges included all motives focused on achieving excitement and stimulation, e.g., the need for competition, curiosity. If discrepancies between judges occurred, the fourth independent judge made the final decision.

### Genotyping

For all subjects, we also performed genotyping for the *DRD4* gene. Genomic DNA was extracted from mucosal swabs with the Swab Extract GeneMATRIX DNA Purification Kit (EURx, Gdansk, Poland). Genotyping was performed by the use of amplified fragment length polymorphism (AFLP). The PCR primer sequences and thermal profiles of the reaction were identical to those published by Dmitrieva et al. (2011). The PCR reaction was conducted in a volume of 20 μl with 0.75 μl (0.75 U) of Color Perpetual Taq DNA Polymerase, 3 μl buffer B, 0.8 μl dNTP mix (5 mM each; EURx, Gdansk, Poland), 1.5 μl DMSO (DNA Gdansk, Poland), 1.5 μl of each primer (10 μM), and 150 ng of genomic DNA. PCR products were visualized on 2% agarose gel stained with SimplySafe (EURx, Gdansk, Poland). This study was carried out in accordance with the recommendations of Ethical Committee of the Medical University of Lublin. All subjects gave written informed consent in accordance with the Declaration of Helsinki. The protocol, the procedures of the study and the genotyping was approved by the Ethical Committee of the Medical University of Lublin.

The results of genotyping revealed that among the successfully genotyped group (*n* = 217), 177 individuals were homozygous (10 were 7+/7+, 167 were 7−/7−) and 40 individuals were heterozygous (7+/7−). Fifty participants (24 investors and 26 non-investors) were classified as 7R+ individuals and 167 participants (89 investors and 78 non-investors) were 7R− individuals. The frequencies of the gene variants (7R+ vs. 7R−) did not differ significantly between groups (*χ*^2^[1, *n* = 217] = 0.43, *p* = 0.511, *φ* = −0.045). Deviations from Hardy-Weinberg equilibrium were determined using the chi-square test. Genotype frequencies were consistent with the Hardy-Weinberg equilibrium (non-investors, *p* = 0.38; investors, *p* = 0.42).

### Statistics

Before the main analysis, we checked the pairwise correlation for the three risk-taking measures that we used (see Table 2). Results revealed that there is: (1) a moderate correlation between stimulating and instrumental risk-taking propensity (*r* = 0.45, *p* < 0.001)—this result is consistent with initial results observed by Zaleśkiewicz (2001); and (2) a weak correlation between the Holt-Laury test and instrumental risk-taking propensity (*r* = 0.18, *p* = 0.009). Thus, we can conclude that the risk-taking measures we used examine different aspects of risk taking.

**Table 2:** Pairwise correlation between risk-taking measures (*p* value in parentheses).

	HLT	SRT	IRT
HLT	1		
SRT	0.111 (0.114)	1	
IRT	**0.178 (0.009)**	**0.448 (< 0.001)**	1

*HLT, risk-taking propensity measured with Holt-Laury test; SRT, stimulating risk-taking; IRT, instrumental risk-taking*.

#### Holt-Laury Test

As we mentioned before, the point at which participant decides to switch from Lottery A to Lottery B can indicate one’s risk preferences. Usually, participants make their decisions in a way that for the first four lottery pairs they prefer Lottery A (it has higher expected value and also guarantees the safer reward), whereas when making decision about the last four lottery pairs, participants prefer Lottery B (it has clearly higher expected value; Holt and Laury, 2002). The crucial point in the Holt-Laury test is a fifth lottery pair at which higher expected value switches form Lottery A to Lottery B. For this pair, expected values of each option are quite similar (Lottery A: 90 vs. Lottery B: 95). Thus one who is risk averse still prefers Lottery A, where risk seeker switches to Lottery B.

What does this tell us about risk preferences? We might conclude that one who chooses Lottery B during the first five lottery pairs is a risk seeker, whereas one who still prefers Lottery A during the last five lottery pair is risk averse. Thus, we analyzed both halves of lottery pairs as separate variables and each participant was checked for two variables: (1) score for Lottery B choices for lottery pairs 1–5; and (2) score for Lottery B choices for lottery pairs 6–10^2^.

To verify if the specific variant of *DRD4* gene (i.e., 7R+) is associated with higher risk-taking propensity measured with the Holt-Laury test and whether it is moderated by experience in financial risk-taking activity (i.e., being an investor or not), we analyzed our data using a 2 (gene: 7R+ vs. 7R−) × 2 (group: investors vs. non-investors) univariate analysis of variance (ANOVA; both factors between-subject), separately for: (1) first half of the test (first five lottery pairs); and (2) second half (last five lottery pairs) as dependent variables.

#### Instrumental Risk-Taking

To assess if the specific variant of *DRD4* gene (i.e., 7R+) is connected with higher instrumental risk-taking propensity (dependent variable) and whether it is moderated by experience in financial risk-taking activity (i.e., being an investor or not), we analyzed our data using a 2 (gene: 7R+ vs. 7R−) × 2 (group: investors vs. non-investors) univariate ANOVA (both factors between-subject).

#### Stimulating Risk-Taking

To verify if the specific variant of *DRD4* gene (i.e., 7R+) is connected with higher stimulating risk-taking propensity (dependent variable) and whether it is moderated by experience in financial risk-taking activity (i.e., being an investor or not), we analyzed our data using a 2 (gene: 7R+ vs. 7R−) × 2 (group: investors vs. non-investors) univariate ANOVA (both factors between-subject).

## Results

### Holt-Laury Test

For the Holt-Laury test, we scored each choice of Lottery B (with higher possible payoff and higher variance) as 1 point. Thus, the ultimate risk-seeker who chose in each pair the riskier lottery could achieve the maximum 10-point score. Taking into account that in the last lottery pair, higher payoffs in both lotteries are certain, we decided to exclude participants (*n* = 19) who chose lottery A in the last pair (with a lower payoff)—we suspect this might suggest that they did not understand the task or answered randomly. Hence, the minimum score in the Holt-Laury test was 1. Eventually, we conducted our analysis on a group of 97 investors and 95 non-investors (six participants did not indicate their choices in each lottery pair).

The results of analysis for first five lottery pairs revealed no significant effects. Neither a main effect of gene (*F*_(1,188)_ = 0.25, *p* = 0.618, $\eta_{\text{p}}^{2}$ = 0.001) nor a main effect of group (*F*_(1,188)_ = 0.15, *p* = 0.695, $\eta_{\text{p}}^{2}$ = 0.001) was significant. As well, we did not observe significant group × sequence (*F*_(1,188)_ = 0.84, *p* = 0.361, $\eta_{\text{p}}^{2}$ = 0.004) interaction.

**Figure 1:**
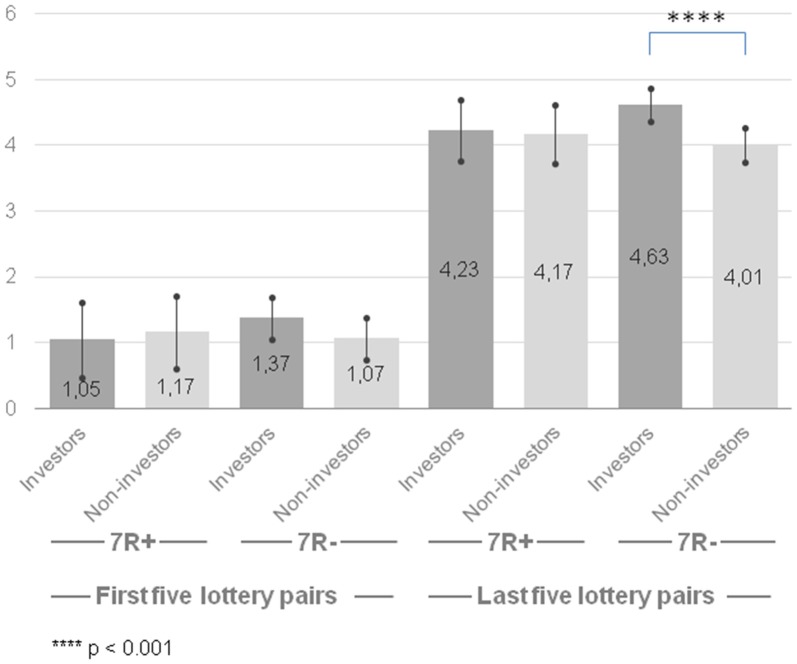
Risk-taking propensity measured with Holt-Laury test for 7R+ and 7R− in the group of investors and non-investors. Higher scores indicate higher risk-taking propensity. Error bars indicate confidence intervals.

The results of analysis for last five lottery pairs revealed that only a main effect of group was marginally significant (*F*_(1,188)_ = 3.24, *p* = 0.074, $\eta_{\text{p}}^{2}$ = 0.017). The group of the investors was more risk-taking (*M* = 4.43, CI [4.16, 4.69] than the group of non-investors (*M* = 4.09, CI [3.83, 4.35]), however this pattern was observed only for 7R− individuals (*F*_(1,188)_ = 11.20, *p* = 0.001, $\eta_{\text{p}}^{2}$ = 0.056). Neither a main effect of gene (*F*_(1,188)_ = 0.44, *p* = 0.510, $\eta_{\text{p}}^{2}$ = 0.002) nor a gene × group interaction (*F*_(1,188)_ = 2.18, *p* = 0.142, $\eta_{\text{p}}^{2}$ = 0.011) was significant (Figure 1).

### Instrumental Risk-Taking

**Figure 2:**
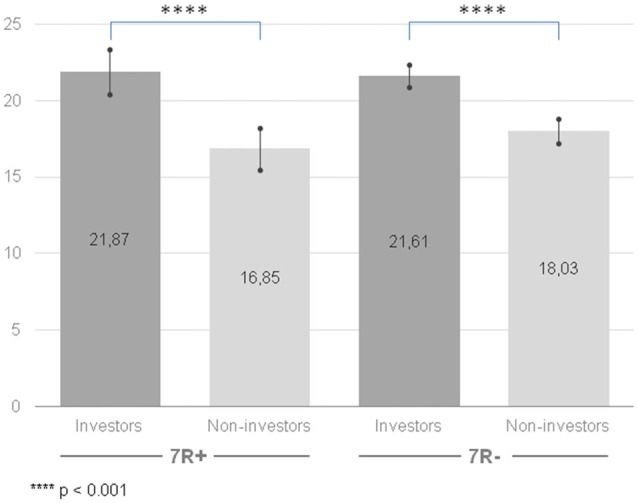
Instrumental risk-taking propensity measured with Stimulating-Instrumental Risk Inventory for 7R+ and 7R− in the group of investors and non-investors. Higher scores indicate higher risk-taking propensity. Error bars indicate confidence intervals.

Once again, we observed a significant difference for a main effect of the group factor (*F*_(1,210)_ = 55.43, *p* < 0.001, $\eta_{\text{p}}^{2}$ = 0.209): the group of investors achieved higher results in instrumental risk-taking propensity (*M* = 21.74, CI [20.92, 22.56]) than the group of non-investors (*M* = 17.47, CI [16.64, 18.23]). The effect existed when investors and non-investors were compared regardless of their *DRD4* gene variant (see Figure 2). However, there were no differences for a main effect of the gene factor (*F*_(1,210)_ = 0.63, *p* = 0.429, $\eta_{\text{p}}^{2}$ = 0.003). The interaction of group × gene was also not significant (*F*_(1,210)_ = 1.561, *p* = 0.213, $\eta_{\text{p}}^{2}$ = 0.007).

### Stimulating Risk-Taking

**Figure 3:**
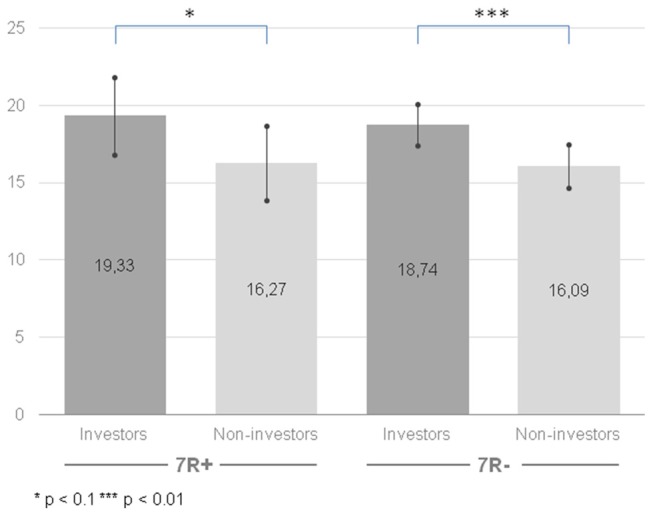
Stimulating risk-taking propensity measured with Stimulating-Instrumental Risk Inventory for 7R+ and 7R− in the group of investors and non-investors. Higher scores indicate higher risk-taking propensity. Error bars indicate confidence intervals.

Similarly, like in the case of instrumental risk-taking, our analysis indicated significant differences for a main effect of the group (*F*_(1,208)_ = 8.022, *p* = 0.005, $\eta_{\text{p}}^{2}$ = 0.037): the group of the investors was more prone to stimulating risk-taking (*M* = 19.04, CI [17.62, 20.45]) than the group of non-investors (*M* = 16.18, CI [14.79, 17.58]). Once again, the effect persisted when comparing 7R− investors (*M* = 18.74, CI [17.41, 20.06]) with 7R− non-investors (*M* = 16.09, CI [14.69, 17.50]; *F*_(1,208)_ = 7.29, *p* = 0.008, $\eta_{\text{p}}^{2}$ = 0.034) and was slightly significant between 7R+ investors (*M* = 19.33, CI [16.83, 21.84]) and 7R+ non-investors (*M* = 16.27, CI [13.86, 18.68]; *F*_(1,208)_ = 3.03, *p* = 0.083, $\eta_{\text{p}}^{2}$ = 0.014; see Figure 3). However, contrary to our hypothesis, we did not observe a main effect of the gene (*F*_(1,208)_ = 0.147, *p* = 0.702, $\eta_{\text{p}}^{2}$ = 0.001). The interaction of group × gene (*F*_(1,208)_ = 0.043, *p* = 0.836, $\eta_{\text{p}}^{2}$ < 0.001) was also not significant.

### Motivation to Engage in Investment Activity

We compared the frequencies of motivation to engage in investment activity between 7R+ and 7R− individuals (stimulating motivation vs. instrumental motivation). Ninety-seven investors indicated an answer to the question about their motivation to engage in investment activity (missing data *n* = 16). Once more, we did not observe a significant difference (*χ*^2^[1, *n* = 97] = 1.35, *p* = 0.245, *φ* = 0.118) between the 7R+ (10 of 22 investors indicated the stimulating motivation) and 7R− individuals (24 of 75 investors indicated the stimulating motivation).

## Discussion

We hypothesized that the previous inconclusive results about the *DRD4* gene might be explained by the moderating role of the motivation to take risk. Namely, if the dopamine gene *DRD4* is associated with a blunted response to dopamine and 7R+ individuals need to seek higher stimulation to feel the same activation in the dopamine reward pathway compared to 7R− individuals, then 7R+ individuals should be more motivated to engage in risky activities that deliver arousal. However, we failed to notice any differences between the 7R+ and 7R− individuals on: (1) the stimulating risk-taking scale; (2) the instrumental risk-taking scale; (3) their indicated motivation to engage in investment activity; and (4) the experimental task—the Holt-Laury test. We observed no differences between neither 7R+ and 7R− investors nor 7R+ and 7R− non-investors. On the other hand, we found evidence that investors are more prone to take risk than non-investors. This result was present for the stimulating and instrumental risk-taking scales (Zaleśkiewicz, 2001). For the Holt-Laury test (Holt and Laury, 2002) we noticed that only 7R− investors were more risk-seeking than non-investors. This might suggest that we used appropriate risk-taking measures, which might distinguish groups with different levels of risk-taking propensity.

Nevertheless, our study is another one to report a lack of differences between 7R+ and 7R− individuals in the domain of financial risk-taking. To our knowledge, our study is the second one that focused on a group of active investors who are experienced in financial decisions and risk-taking. In a previous study, conducted by Anderson et al. (2015), a sample of 140 active investors were examined, and there was no significant relationship between the *DRD4* gene and risk-taking in three risk-taking measures: measures of equity holdings, multiple price listing, and the survey risk measure. Also, Dreber et al. (2012) failed to find differences between 7R+ and 7R− when the subject pool was composed of professional decision-makers (i.e., owners, presidents, and managers of large companies). Only one study (Dreber et al., 2011) where participants were not undergraduate students noticed a significant association between the 7R+ variant and risk-taking (see Table 3 for a summary of previous results and tested subject pool). These findings and our results might suggest it is likely that the relationship of the *DRD4* gene with risk-taking is mediated by environmental factors, e.g., experience, familiarity with risky situations, or wealth. For example, Lo and Repin (2002) demonstrated that during live trading sessions, the autonomic responses of more experienced investors were significantly lower than less experienced traders. It is possible then that the level of experience among our subject pool was heterogeneous, and, thus, a few factors were associated with lower emotional reactions, not only the specific variant of the *DRD4* gene. This might be a reason why the 7R+ and 7R− investors did not differ in risk-taking propensity.

**Table 3:** Summary of the existing studies on the *DRD4* gene and risk-taking propensity.

Reference	Subject pool	Group size	Risk-taking measures	Result
Dreber et al. (2009)	Undergraduate students	94 (7R+ *n* = 24)	Experimental investment task	7R+ more risk-taking
Kuhnen and Chiao (2009)	Undergraduate students	65 (7R+ *n* =15)	Experimental investment task	7R+ more risk-taking
Carpenter et al. (2011)	Mainly undergraduate students (*n* = 125)	140 (7R+ *n* = 51)	Three gambling tasks—lottery choices with: Known probabilities Ambiguous probabilities Possible loss	No differences 7R+ more risk-taking 7R+ more risk-taking (*p* = 0.10)
Dreber et al. (2011)	Bridge players	98 men (7R+ *n* = 16) 77 women (7R+ *n* = 6)	Bridge risk-taking Experimental investment task	7R+ more risk-taking only among men in both measures
Eisenegger et al. (2010)	No info, mean age 23.5 (SD = 3.6)	200 (7R+ *n* = 42)	Gambling task	No differences in control (placebo administration) group
Frydman et al. (2011)	Undergraduate students	90 (no info)	Gambling task	No differences
Dreber et al. (2012)	Owners, presidents, and managers of large companies	121 (7R+ *n* = 17)	Experimental investment task	No differences
Anderson et al. (2015)	Investors	149 (7R+ *n* = 53)	Measures of equity holdings Multiple price listing Survey risk measure (Dohmen et al., 2011)	No significant differences

We are cautious with interpreting our results and do not claim that there is no relationship between the *DRD4* gene and risk-taking. There are numerous studies demonstrating that genes may determine risk preferences (e.g., Cesarini et al., 2010; Cronqvist and Siegel, 2014) and also a few studies have revealed that 7R+ individuals take more risks than 7R− individuals (Dreber et al., 2009, 2011; Kuhnen and Chiao, 2009; Carpenter et al., 2011). Nevertheless, as Benjamin et al. (1996) observed on a group of almost 10,000 subjects, the single nucleotide polymorphism across the human genome can explain a maximum 1.25% variation of any psychological trait. Moreover, the association of the *DRD4* gene and risk taking is probably a complex phenomenon and the risk-taking trait in general depends on many factors, such as individual differences, sex, age, financial knowledge, income and cognitive abilities (Hallahan et al., 2003; Bali et al., 2009; Burks et al., 2009; Mayfield and Shapiro, 2010).

Our present study reveals that the type of motivation (i.e., stimulating and instrumental) underlying the risk-taking activity is not a factor that mediates the relationship between *DRD4* and risk taking. Perhaps our main finding is evidence that 7R+ individuals might be highly heterogeneous. As we observed, 7R+ investors were significantly more prone to risk-taking than 7R+ non-investors. To our knowledge, this is the first study that reports differences between two groups of 7R+ individuals and gives strict evidence that the variation in risk-taking among 7R+ individuals is environmentally sensitive and might depend on factors like familiarity with financial risky decision-making, i.e., being an investor or not.

Of course, our study has limitations. As one of the risk-taking measures, we used the Holt-Laury test with only hypothetical payoffs. This could be perceived by our subjects (especially investors) as not engaging and thus induce responses not convergent with real-life risk-taking propensity. However, as Holt and Laury (2002) indicated, using high hypothetical payoffs (as in our study) elicits the proper level of risk aversion. Moreover, as Camerer and Hogarth (1999) noticed on the basis of 74 studies with no, low, or high real payoffs, the presence of monetary incentives does not influence the mean performance. Thus, we believe that the level of risk-taking propensity measured with the Holt-Laury test was not affected by the lack of possible winnings. Another possible limitation is that we used a questionnaire scale to assess the stimulating and instrumental risk-taking propensity. Due to self-reported estimations that highly rely on self-perception, subjects could not accurately present their real behaviors. For example, Brañas-Garza et al. (in press) observed using a large sample that the digit ratio (2D:4D—a biomarker for prenatal testosterone exposure) was significantly associated with risk preferences; however, this was noticed when risk-taking propensity was measured by the experimental task. There was no relationship between 2D:4D and risk-taking propensity as measured by the self-reported scale. As Brañas-Garza et al. (in press) noticed, this result could arise because of the complexity of risk-taking behavior and the fact that various risk-taking measures correlate only imperfectly. However, in our study, we observed a lack of differences not only in self-reported risk-taking propensity but in the experimental task as well. Moreover, we used measures that examine different nuances of risk taking—we observed only a moderate correlation between stimulating and instrumental risk-taking scales and a weak correlation between instrumental risk-taking and the Holt-Laury test. All of this suggests that the lack of differences between 7R+ and 7R− individuals in our study is not a case of inadequate selection of methods but is rather a robust finding. Also, the lack of differences in motivation for engaging in investment activity between 7R+ and 7R− investors seems to be in line with the above assumption. As we mentioned before, asset allocation in the stock market is a risky activity itself. Thus, if the 7R+ individuals should seek more stimuli to overcome the blunted response to dopamine, we should expect that they would be more willing to engage in investment activity because for reasons of stimulation. However, one more time we observed no differences between 7R+ and 7R− individuals, which supports previous results.

As previous studies revealed inconclusive results about the association between the DRD4 gene and risk-taking, it is worth wondering whether this relation might be moderated by some other psychological factors than instrumental and stimulating risk-taking. For example, if 7R+ are more risk-taking due to the need for stimulation and seeking for positive feelings, it is possible that individual differences in susceptibility to affect might moderate this relation. Consider a 7R+ individual who is not sensitive to changes in affect—we can imagine that in such a case two factors might work in opposite directions: the 7R+ variant increases the need for stimuli, whereas the lack of susceptibility to affect attenuates this impact. Thus, changes in arousal and emotional states might not have an impact on the behavior of individuals with low susceptibility to affect (a 7R+ individual). In our study, we wanted to avoid the issues related to multiple testing and thus, we decided to focus only on two psychological factors: instrumental and stimulating risk-taking. Hence, this explanation is only hypothetical and needs further investigation.

Moreover, in our study we focused solely on psychological factors that could potentially mediate relation between *DRD4* gene and risk-taking; and as previous studies revealed (e.g., Docherty et al., 2012) also epigenetic processes associated with e.g., methylation levels at the promoter of the *DRD4* gene may mediate genetic influences. It was revealed that methylation levels at the promoter of the *DRD4* gene are associated with schizophrenia (Cheng et al., 2014), Alzheimer’s disease (Ji et al., 2016), drug addiction (Ji et al., 2018) and alcohol dependence (Zhang et al., 2013). Thus, future work is needed to verify if other than psychological factors (e.g., methylation levels) might also mediate the relation between the *DRD4* gene and financial risk-taking.

It is also worth noting that our procedure included only tasks that probably did not induce the feelings of excitement or stimuli. Perhaps a procedure with tasks that elicit arousal is needed to catch the differences between the 7R+ and 7R- individuals in the domain of financial risk-taking. A similar procedure with “cold” (less emotional) and “hot” (much more arousing) tasks were used by Costa et al. (2014) to examine the impact of a factor that might potentially decrease emotional arousal on decision-making. What occurred was that in the case of the “hot” version, significant differences were observed. The “cold” one revealed no significant results.

At the end, note that in our study we used the traditional procedure of Holt-Laury test that is, we presented items in a fixed order starting with a very low probability of winning a higher prize that increased in subsequent lottery pairs. Such sequence could suggest the strategy of choices based on a need for consistency to avoid cognitive dissonance (Festinger, 1957): “If I chose riskier lottery (Lottery B) in the earlier pair I would also do the same in a next step (when Lottery B is less risky)”. It is possible that participants, especially investors who are familiarized with financial decision making, noticed such linear sequence what could potentially influence their choices. Thus, it would be beneficial to test how subjects respond to Holt-Laury test when presenting the items in a random way.

In sum, we still need more research to better understand the genetic foundations of risk-taking, which could answer the question about the substantial variation in the domain of risky financial decisions. However, it seems that we need to examine homogeneous groups, i.e., undergraduate students, if we want to observe substantial differences. Otherwise, the effect of the genes might be suppressed by environmental factors.

## Author Contributions

RM, MK and MG conceived the study. RM, MK, MM-W, MG, AF, PG and PM designed the study protocol. RM drafted the manuscript, coordinated the data gathering, and carried out the statistical analyses. MK, MM-W and MG helped draft the manuscript. MM-W conducted the genetic analyses. AF, PG and PM revised the manuscript. AF provided advice and facilities for genetic analyses. All authors gave final approval for publication.

## Conflict of Interest Statement

The authors declare that the research was conducted in the absence of any commercial or financial relationships that could be construed as a potential conflict of interest.

## Footnotes

^1^This result is robust, controlling for gender and overconfidence.

^2^We would like to thank to our Reviewer for suggesting this analysis.
